# High Incubation Temperature and Threonine Dietary Level Improve Ileum Response Against Post-Hatch *Salmonella* Enteritidis Inoculation in Broiler Chicks

**DOI:** 10.1371/journal.pone.0131474

**Published:** 2015-07-01

**Authors:** Alexandre Lemos de Barros Moreira Filho, Celso José Bruno de Oliveira, Heraldo Bezerra de Oliveira, Danila Barreiro Campos, Ricardo Romão Guerra, Fernando Guilherme Perazzo Costa, Patricia Emília Naves Givisiez

**Affiliations:** 1 Department of Animal Science, Center of Agrarian Sciences, Federal University of Paraiba (UFPB), Areia, PB, Brazil; 2 Department of Veterinary Sciences, Center of Agrarian Sciences, Federal University of Paraiba (UFPB), Areia, PB, Brazil; Indian Institute of Science, INDIA

## Abstract

This study assessed the effect of both embryonic thermal manipulation and dietary threonine level on the response of broilers inoculated with *Salmonella* Enteritidis, considering bacterial counts in the cecal contents, intestinal morphology, mucin and heat shock protein 70 gene expression, body weight and weight gain. Thermal manipulation was used from 11 days of incubation until hatch, defining three treatments: standard (37.7°C), continuous high temperature (38.7°C) and continuous low temperature (36.7°C). After hatch, chicks were distributed according to a 3x2+1 factorial arrangement (three temperatures and two threonine levels and one sham-inoculated control). At two days of age, all chicks were inoculated with *Salmonella* Enteritidis, except for the sham-inoculated control group. There was no interaction between the factors on any analyses. High temperature during incubation was able to reduce colonization by *Salmonella* Enteritidis in the first days, reducing both *Salmonella* counts and the number of positive birds. It also increased mucin expression and decreased Hsp70 expression compared with other inoculated groups. High temperature during incubation and high threonine level act independently to reduce the negative effects associated to *Salmonella* Enteritidis infection on intestinal morphology and performance, with results similar to sham-inoculated birds. The findings open new perspectives for practical strategies towards the pre-harvest *Salmonella* control in the poultry industry.

## Introduction

Salmonellosis is a worldwide foodborne disease and remains as a major concern for the global food trade [1]. *Salmonella enterica* serovar Enteritidis (SE) is amongst the leading serovars causing salmonellosis in humans [2–4], and it is mostly associated with the consumption of poultry products such as eggs and meat [5].

Pre-harvest *Salmonella* control is considered a key aspect for the reduction of the disease in humans. Therefore, on-farm measures are important to reduce the *Salmonella* carriage at slaughter and consequently cross-contamination to carcasses. SE was the most isolated serovar from poultry carcasses in United States abattoirs [6].

Birds are more susceptible to *Salmonella* contamination in the first days of life [4], and during this period the intestinal microbiota is not established yet. Commensal bacterial populations in the intestine protect the host from the colonization by pathogenic bacteria [7], since they compete for binding sites on the epithelium and for available nutrients; they also produce bacteriocins and strengthen the intestinal immune response [8]. Mucin has an important role on this response, favoring the establishment and maintenance of a comensal microbiota, and forming a protection barrier against pathogens. Mucin production is intimately correlated to threonine levels in the diet, which have been associated with protection of the intestinal epithelium [9]. Therefore, threonine has been considered to be an important nutrient for the defense against intestinal pathogens in birds and swine [9,10]. Threonine is involved in important biological functions such as the maintenance, integrity and immunity of the intestinal mucosa and, consequently, dietary requirements may change according to the importance of each function [11].

Thermal manipulation during embryogenesis is suggested as an alternative to improve bird responses to stress situations, mainly heat stress, later in life [12–15]. Physiologically, early thermal manipulation induces epigenetic and metabolic mechanisms that control body temperature in the long term. Further, it has the advantage of enhancing poultry resistance to environmental changes without much effect on growth performance [15]. Since the stress response is non-specific, thermotolerance induction might also improve resistance to other kinds of stresses. In fact, non-infectious stress situations may prepare the immune system to fight against infectious challenges [16,17]. Although the consequences of thermal manipulation during incubation on the performance and meat quality of broiler chickens have been widely assessed, the responses of birds to other types of stresses have not been addressed to date.

Our hypothesis is that thermal manipulation during incubation may interfere with post-hatch resistance of chicks and interact with threonine dietary levels, improving the responses of broilers to a challenge with *Salmonella* Enteritidis at 2 days of age. We found that high incubation temperature was able to decrease the number of SE-colonized birds and SE counts in the positive birds. Furthermore, both high dietary threonine and high incubation temperature act independently and improve ileum morphometry after SE challenge, increase goblet cell counts and MUC2 mRNA expression and decrease Hsp70 mRNA expression in chicks, which resulted in better performance at 10 days of age.

## Material and Methods

### Experimental design and general management

All management, slaughter and sampling procedures were approved by the Ethical Committee of Animal Use in Research of the Federal University of Paraíba (Comissão de Ética no Uso de Animais da Universidade Federal da Paraíba) under the protocol number CEUA 0311/13.

Two hundred and forty eggs were obtained from a commercial hatchery (Guaraves Alimentos Ltda, Guarabira, PB, Brazil), with mean weight of 69.0 ± 2.95 g. The eggs, originated from Cobb500 breeders aged 44 weeks, were distributed into three artificial incubators (IP130, Premium Ecológica Ltda, Belo Horizonte, MG, Brazil). All incubators were kept under standard incubation conditions from one to eleven days (D11) at 37.7°C and 60% relative humidity, and automatic turning at each two hours. On D11, eggs were candled and dead embryos or white eggs were discarded. Temperatures were set to 37.7°C (standard group), 38.7°C (continuous high temperature) and 36.7°C (continuous low temperature) from D11 to hatch.

At hatch, chicks were identified with leg bands and individually weighed. Twenty birds with mean body weight similar to the mean of all birds (50.7 ± 5.1 g) were randomly selected from each incubation treatment and cloacal swabs were sampled for *Salmonella* screening from 10 birds per incubation temperature. The birds were then distributed into a completely randomized experimental design according to a factorial 3x2+1, with three incubation temperatures (standard, high and low) and two levels of threonine in the diet (basal and high) and a sham-control group comprised of standard-temperature incubated birds fed the diet with basal threonine level. Thus, there were seven treatments with 10 birds (repetitions) kept into boxes. Each box was equipped with feeder and drinker and was covered with nylon to avoid contamination between boxes by vectors such as flies. Thermo-hygrometers (Oregon Scientific, Portland, EUA) were used to monitor temperature and relative humidity in the room.

Basal diets (S1 Table) were corn-soybean meal based and formulated according to Rostagno et al. [18]. Levels were 22.16% CP, 2,950 kcal/kg ME, 1.31% digestible lysine, 0.94% digestible methionine + cystine and 0.857% digestible threonine in the pre-initial phase (1 to 7 d). Initial phase basal diet contained 20.80% CP, 3,002 kcal/kg ME, 1.18% digestible lysine, 0.85% digestible methionine + cystine and 0.764% digestible threonine. Threonine level was 12% higher in high-threonine diets compared to the basal levels (0.956% in the pre-initial and 0.852% in the initial phase); other levels were similar.

Birds were weighed individually at the beginning and in the end of the experimental period. Initial and final body weights were used to calculate weight gain.

### Inoculation

*Salmonella* challenge was performed when birds were two days old. Inoculum was prepared using one colony of nalidixic-acid resistant *Salmonella* Enteritidis (SE^Nal+^) into nutrient broth (Acumedia, USA) containing nalidixic (100 μg/mL) acid for 24h at 37°C and then an aliquot (0.1 mL) was cultured again for four hours at 37°C. Inoculum concentration was determined by plating a serial dilution (10^-1^ to 10^-6^) onto brilliant green agar plates (Acumedia, USA) with nalidixic acid (100 μg/mL) and incubating at 37°C. *Salmonella* colonies were counted after 24 hours. All birds in each box were inoculated with 0.5mL of *Salmonella* (9.5 x 10^7^ UFC/mL) into the crop, except sham-inoculated control birds, which were given only nutrient broth.

### Microbiological analysis

At ten days of age (8 days post-inoculation, dpi), all birds were weighed and slaughtered by cervical dislocation. Cecal contents were collected from all birds of each treatment (n = 10) and weighed before being serially diluted (10^-1^ to 10^-6^) with peptone water (Acumedia, USA). Twenty-microliter aliquots were cultured in brilliant green agar with nalidixic acid (100 μg/mL) and incubation at 37°C for 24 hours. Colony counts were performed and values were expressed into colony forming units per gram of cecal content (CFU/g).

### Histology

Four birds per treatment were slaughtered and the medial region of the ileum was sampled for histology analyses. Samples were rinsed with sterile saline, immersed in buffered formalin for 24 hours and rinsed in 70% ethanol. Slides were mounted with 5μm sections and stained with hematoxylin-eosin. Two slides with five to seven sections were mounted per bird. Photomicrographs of the duodenal mucosa were taken using a digital camera with 12.1 megapixels (Sony Inc.) connected to a light microscope, with 1.7 optical zoom and 10X-objective. The images were analyzed with Image J software [19]. Villus height, crypt depth and villus:crypt ratio were determined. Villus height was measured from its apex to the basal region, crypt depth was measured from the crypt basis to the region of transition between the villus and crypt. For each animal, 30 measurements were taken, with a total of 120 measures per treatment per parameter. Ratio was calculated between villus height and crypt depth.

Goblet cell counts were performed in sections stained with Periodic Acid-Schiff (PAS) using a analysis software (Motic Image Plus 2.0, Motic, China) in 2000μm-linear segments of 12 repetitions per treatment (three photomicrographs from each of four slides).

### MUC2 and Hsp70 mRNA expression

Total RNA was isolated from three ileum samples per treatment (500 mg) using the RNAspin Mini RNA Isolation Kit (GE Healthcare) according to the manufacturer’s guidelines. Concentration and purity were determined at 260/280 and 260/230 using spectrophotometer (NanoDrop 2000, Thermo Scientific, Wilmington, DE). Reverse transcription was performed with AffinityScript QPCR cDNA Synthesis Kit (Agilent Technologies) according to the manufacturer’s guidelines.

Relative quantification in real time polymerase chain reaction (PCR) was performed using the Brilliant III Ultra-Fast SYBR QPCR Master Mix (Agilent Technologies) according to the manufacturer’s guidelines. Cycling was carried out in a Stratagene Mx3005P (Agilent Technologies). MUC2 primer sequences were 5’-ATGTTTTTGCATCCCATTGC-3’ (forward) and 5’-TGCGGTGGATTGTCAGAATA-3’ (reverse), Hsp70 primer sequences were 5’-GGCTGGAGAGAAGAATGTGC-3’ (forward) and 5’-CAGCTGTGGACTTCACCTCA -3’ (reverse) and β-actin primer sequences were 5’-ACCACTGGCATTGTCATGGACTCT-3’ (forward) and 5’-TCCTTGATGTCACGGACGATTTCC-3’ (reverse). Primers were designed using Primer Express (version 3, Applied Biosystems, Foster City, CA).

Relative MUC2 and Hsp70 mRNA abundance was determined using the method 2^-ΔΔ^Ct [20]; Ct values of each sample were standardized for β-actin RNA.

### Statistical analyses

All data of inoculated birds were evaluated according to a completely randomized design in a factorial arrangement (three incubation temperatures and two threonine dietary levels). Microbiological and performance (initial weight, final weight and weight gain) data were analyzed using ten repetitions; morphometric data comprised 30 measurements per animal per parameter (120 repetitions per treatment); and goblet cell counts were assessed with three counts per animal and a total of 12 repetitions per treatment. Data were submitted to ANOVA and means were compared by the Tukey’s test at 5% probability. The sham-inoculated control was compared with each of the inoculated treatments in the morphometric analysis and goblet cell counts using Dunnet test at 5% of probability. Fisher’s exact test at 5% probability was used to compare the number of SE-positive birds between treatments during incubation or between threonine levels.

## Results

There was no interaction (p>0.05) between incubation temperature and threonine levels on any parameter; therefore, the means for the main factors in microbiological (Table 1), performance (Table 2) and morphology are presented (Table 3, S2 Table). Incubation parameters and chick weight were not affected by the thermal manipulation from D11 to hatch (S3 Table).

**Table 1:** *Salmonella* counts in the cecal contents (Log_10_ CFU/g) and number of colonized (positive) birds (8dpi) submitted to thermal manipulation during incubation and fed different threonine levels post-hatch (n = 10 per treatment).

		Mean CFU/g cecal content (Log_10_)^1^	Colonized (positive birds)/ Total of birds
Incubation temperature	Low (36.7°C)	5.20 ± 2.14 a	20/20 A (100%/100%)
	Standard (37.7°C)	5.15 ± 2.12 a	19/20 A (95%/100%)
	High (38.7°C)	2.72 ± 1.12 b	11/20 B (55%/100%)
Threonine level	Basal (0.857%)	4.78 ± 1.97 a	27/30 A (90%/100%)
	High (0.956%)	3.93 ± 1.62 a	23/30 A (77%/100%)
Significance level	Temperature (Y)	< 0.01	
	Threonine (Z)	ns	
	Interaction (YxZ)	ns	

Within each factor, means followed by similar small letters in the column are similar by Tukey's test (5%). Different capital letters indicate difference by the Fisher's exact test (p = 1.0 between Low/Standard; p = 0.0012 between Low/High; p = 0.0084 between Standard/High; p = 0.29 between Basal/High threonine).

^1^Chicks were inoculated with *Salmonella* Enteritidis at 2 days of age. Sham-inoculated control was incubated at standard incubation temperature and fed basal threonine levels, and showed no counts at 10 days of age.

**Table 2:** Initial weight (1d), final weight (10d) and weight gain of chicks submitted to thermal manipulation during incubation and fed different threonine levels post-hatch (n = 10 per treatment).

		Initial weight (g)	Final weight (g)	Weight gain (g)
Incubation temperature	Low (36.7°C)	50.9 ± 1.9 aA	220.3 ± 17.4 aB	169.4 ± 15.7 aB
	Standard (37.7°C)	50.5 ± 2.8 aA	223.5 ± 15.8 aA	172.9 ± 13.2 aA
	High (38.7°C)	50.4 ± 2.6 aA	227.9 ± 15.9 aA	177.5 ± 13.6 aA
	Sham-inoculated^1^	51.0 ± 2.8 A	236.1 ± 18.1 A	185.1 ±15.3 A
Threonine level	Basal (0.857%)	50.4 ± 2.5 aA	219.9 ± 15.6 aB	169.6 ± 13.4 aB
	High (0.956%)	50.8 ± 2.4 aA	227.8 ± 15.2 aA	177.1 ±13.0 aA
	Sham-inoculated	51.0 ± 2.8 A	236.1 ± 18.1 A	185.1 ±15.3 A
Significance level	Temperature (Y)	ns	ns	ns
	Threonine (Z)	ns	ns	ns
	Interaction (YxZ)	ns	ns	ns

Within each factor, means followed by the same small letter in the column are similar by Tukey’s test (5%).

Within each factor, means followed by the same capital letter in the column are similar to the sham-inoculated treatment by Dunnet’s test (5%).

^1^Chicks were inoculated with *Salmonella* Enteritidis at 2 days of age, except for the sham-inoculated control group. Sham-inoculated control was incubated at standard incubation temperature and fed basal threonine levels.

**Table 3:** Ileum morphology (n = 120 per treatment) and goblet cell counts (n = 12 per treatment) of chicks submitted to thermal manipulation during incubation and fed different threonine levels post-hatch.

		Villus height (V, μm)	Crypt depth (C, μm)	V:C ratio (μm/μm)	Goblet cells (n)
Incubation temperature	Low (36.7°C)	504.3 ± 38.9 bB	63.8 ± 8.7 aA	8.1 ± 0.5 cB	148.3 ± 28.6 aA
	Standard (37.7°C)	491.9 ± 40.8 bB	57.4 ± 7.9 bA	8.7 ± 0.6 bB	152.8 ± 24.8 aA
	High (38.7°C)	551.8 ± 50.6 aA	54.3 ± 7.5 cA	10.3 ± 0.6 aA	164.2 ± 30.5 aA
	Sham-inoculated^1^	575.3 ± 34.5 A	37.0 ± 7.4 B	10.2 ± 0.7 A	123.3 ± 13.7 B
Threonine level	Basal (0.857%)	512.1 ± 39.5 bB	58.7 ± 8.1 aA	8.9 ± 1.1 bB	138.5 ± 23.0 bB
	High (0.956%)	523.3 ± 56.2 aB	58.3 ± 8.7 aA	9.2 ± 0.9 aB	171.7 ± 25.6 aA
	Sham-inoculated	575.3 ± 34.5 A	37.0 ± 7.4 B	10.2 ± 0.7 A	123.3 ± 13.7 B
Significance level	Temperature (Y)	< 0.01	< 0.01	< 0.01	ns
	Threonine (Z)	< 0.01	ns	< 0.01	< 0.05
	Interaction (YxZ)	Ns	ns	ns	ns

Within each factor, means followed by the same small letter in the column are similar by Tukey’s test (5%).

Within each factor, means followed by the same capital letter in the column are similar to the sham-inoculated treatment by Dunnet’s test (5%).

^1^Chicks were inoculated with *Salmonella* Enteritidis at 2 days of age, except for the sham-inoculated control group. Sham-inoculated control was incubated at standard incubation temperature and fed basal threonine levels.

Bacterial counts at 8 days post-inoculation (dpi) of the birds infected with SE^Nal+^ are shown in Table 1. Sham-inoculated control birds showed no *Salmonella* counts. Considering incubation temperature, birds submitted to high temperature during incubation (38.7°C) had lower bacterial counts (p<0.01) when compared with the birds of other treatments (Table 1). The number of inoculated birds that became positive was lower in the heat-treated birds (55%, p<0.01) compared with the other incubation treatments, indicating that the continuous high temperature during incubation was able to reduce the colonization of SE-inoculated birds. On the other hand, all cold-treated and inoculated birds (100%) showed SE^Nal+^ counts. It should be noted that, independent of the number of birds that had SE counts, heat-treated birds that were positive for *Salmonella* showed lower bacterial count values when compared with the birds of other treatments. Considering threonine levels, both SE^Nal+^ counts (4.68 vs 3.93; p = 0.0692) and the number of positive animals (77 vs 90%, p = 0.29) were similar between high and basal levels.

Initial weight, final weight and weight gain (Table 2) showed no interaction between incubation temperature and threonine levels, and there was also no effect of the main factors (p>0.05). On the other hand, when birds incubated at different temperatures and inoculated are compared with sham-inoculated birds, low-temperature incubated birds showed lower final weight and weight gain (p<0.05, Dunnet). Birds incubated at standard or high temperature had similar performance to sham-inoculated birds. As for threonine levels, birds fed with the basal level and inoculated had lower weight gain and final weight (p<0.05) compared to sham-inoculated birds. Birds fed higher threonine level and sham-inoculated birds had similar performance (p>0.05).

Villus height, crypt depth, villus:crypt ratio and goblet cell counts in the ileum are shown in Table 3. Birds that had been incubated at high temperature had better results for villus height, crypt depth and villus:crypt ratio. Low-temperature birds had deeper crypts and smaller villus:crypt ratio (p<0.05). As for threonine levels, increased villus height and smaller crypt depth (p<0.05) were seen with higher threonine supplementation that resulted in higher villus:crypt ratio compared with birds fed basal threonine levels.

Sham-inoculated birds and inoculated high-temperature birds showed better (p<0.05) results for villus height and villus:crypt ratio when compared to inoculated birds that were incubated in standard or low temperatures. Crypt depth was greater (p<0.05) in inoculated birds compared with the sham-inoculated group. Similar results were seen in the jejunum, but not in the duodenum (S2 Table). Independent of threonine level in the diet, birds that were inoculated showed smaller villus height, deeper crypts and lower villus:crypt ratio when compared to sham-inoculated controls (Table 3). This was similar in the duodenum and jejunum, except that villus height in the jejunum was similar between sham-inoculated birds and those inoculated and fed diets with different threonine (S2 Table).

There was no effect of incubation temperature on goblet cell counts (Table 3), but higher threonine dietary level increased the number of goblet cells (p<0.05). Counts were also higher in inoculated than in sham-inoculated birds, independent of incubation temperature, whereas inoculated birds fed higher threonine levels showed higher counts when compared with sham-inoculated birds. Jejunum results were similar to ileum, but counts differed in the duodenum (S2 Table).

**Fig 1:**
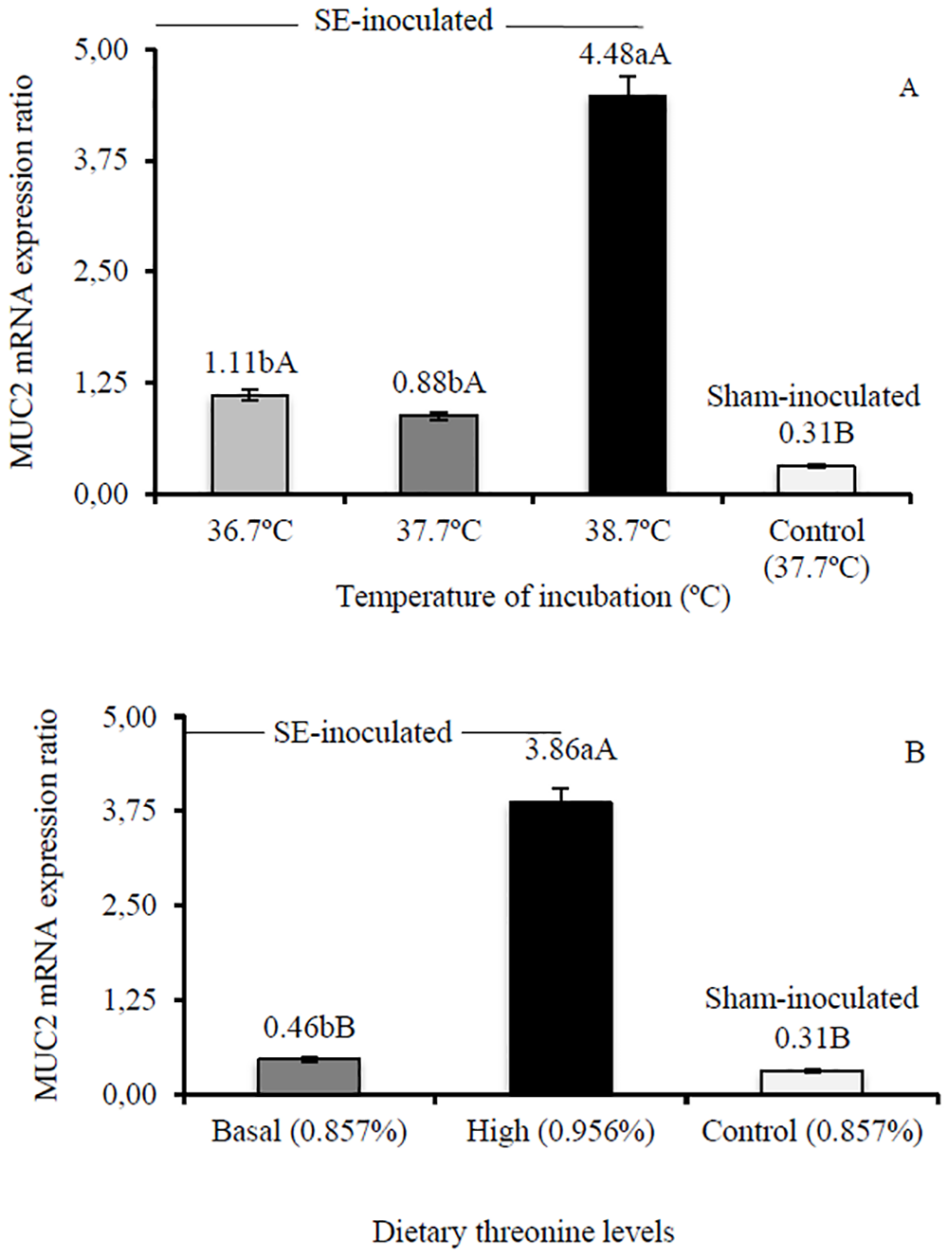
Ileum mucin 2 (MUC2) mRNA of chicks at 10 days of age (n = 3 per treatment). Changes in MUC2 mRNA expression are normalized to β-actin mRNA and expressed relative to the reference gene. Means followed by the same small letter are similar by Tukey’s test. Means followed by the same capital letter are similar to the sham-inoculated treatment (Control) by Dunnet’s test (5%). SE-inoculated, inoculated with *Salmonella* Enteritidis at 2 days of age; sham-inoculated, inoculated with nutrient broth at 2d. (A) Effect of thermal manipulation during incubation. (B) Effect of post-hatch threonine dietary levels.

**Fig 2:**
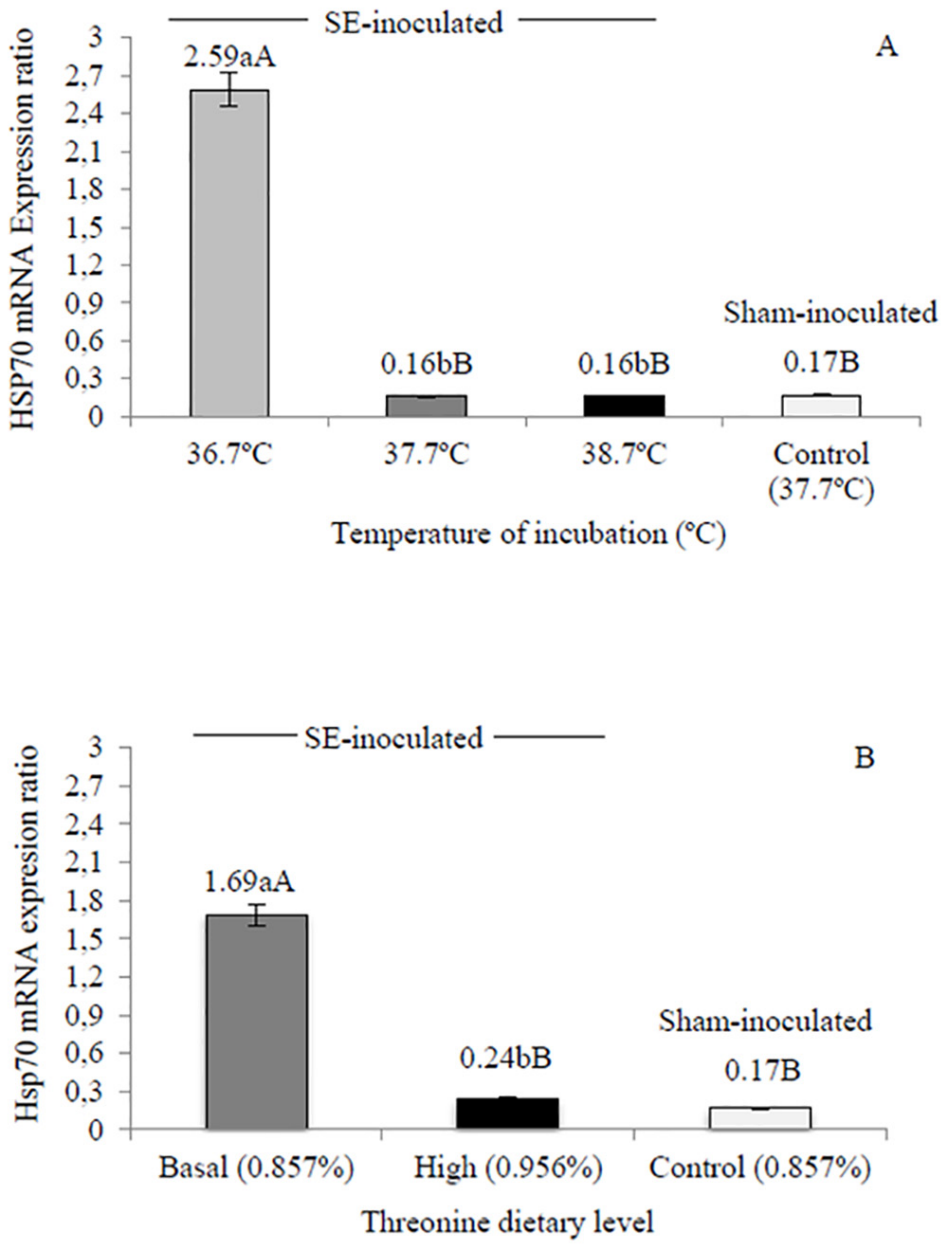
Ileum heat shock protein 70 kDA (Hsp70) mRNA of chicks at 10 days of age (n = 3 per treatment). Changes in Hsp70 mRNA expression are normalized to β-actin mRNA and expressed relative to the reference gene. Means followed by the same small letter are similar by Tukey’s test. Means followed by the same capital letter are similar to the sham-inoculated treatment (Control) by Dunnet’s test (5%). SE-inoculated, inoculated with *Salmonella* Enteritidis at 2 days of age; sham-inoculated, inoculated with nutrient broth at 2d. (A) Effect of thermal manipulation during incubation. (B) Effect of post-hatch threonine dietary levels.

In the current study, expression of MUC2 mRNA was increased in infected birds compared to sham-inoculated birds, and the effect is statistically significant (p<0.05) in the birds incubated in high temperature (14.45 fold) or fed high threonine levels (12.45 fold, Fig 1). Amongst inoculated birds, high-temperature incubated birds showed higher expression of MUC2 mRNA compared to other incubation temperatures (Fig 1A) and high-threonine fed birds showed higher expression than basal-threonine fed birds (Fig 1B). Hsp70 mRNA was similar (p>0.05) in high-temperature and high-threonine inoculated birds when compared to sham controls, but increased in low-temperature (15.23 fold) and basal-threonine birds (9.94 fold, Fig 2). Amongst inoculated birds, low-temperature incubated birds showed higher expression of Hsp70 mRNA compared to other incubation temperatures (Fig 2A) and basal-threonine fed birds showed higher expression than high-threonine fed birds (Fig 2B).

## Discussion

The protective effects of high incubation temperature against early colonization by *Salmonella* Enteritidis in chicks are intriguing and have not been reported to date. Although not presenting as direct a relationship with intestinal mucosa protection as threonine has, high incubation temperature resulted in responses similar to those of high-threonine fed birds concerning MUC2 expression and intestinal integrity. Furthermore, all those responses correlated with better performance at 10 days of age in the high-temperature incubated group, which also presented lower number of positive animals.

Hsp70 synthesis has been suggested as a key mechanism associated to increased resistance against *Salmonella* and to infectious bursa disease in post-hatch heat-stressed birds also submitted to a second stressor, i.e., feed restriction [21,22]. Our results, however, indicate that Hsp70 expression is not correlated to higher resistance against *Salmonella* in heat-stressed chicks during incubation, since the levels of Hsp70 expression in those animals were similar to those incubated at thermoneutral conditions, which were more susceptible to colonization. On the other hand, low incubation temperature has not contributed for the resistance and birds showed a more evident stress response, with higher levels of Hsp70 mRNA.

It has been suggested that exposition to non-infectious stressful situations might prepare the immune system to fight against infectious challenges [16,17] and protection mediated by heat shock proteins against inflammatory and infectious processes occurs due to the suppression of inflammatory mediators [23]. Indeed, lower stress response with reduction in the levels of corticosterone has been reported in three-old-chicks previously treated with heat between D16 and D18 [24].

On the other hand, our results suggest that temperature manipulation during incubation apparently resulted in an epigenetic effect that endured during the first days post-hatch and might have contributed to the higher resistance to *Salmonella* infection in the case of high temperature. We strongly believe that such protective effect might not be due to the inflammatory axis only, but might also involve complex metabolic issues. For instance, accelerated yolk sac absorption may have resulted from the faster metabolism of chicks during incubation in high temperature [13] and from increased glucose needs before hatch and during the hatching process [25,26]. Yolk sac absorption is determining to the proper development of the gastrointestinal tract in the chick in the first 48 h after hatch [27], the same age used in the present study for SE challenge.

It is important to mention that our findings do not corroborate previous studies that reported negative effects of high incubation temperatures on hatchability, embryo mortality, and chick weight [13,28]. On the contrary, high temperature resulted in higher hatchability and similar chick weights compared with the other temperatures (S3 Table), corroborating other previous studies [12,29].

Threonine levels had no effect on *Salmonella* counts in the present study or the number of infected birds (Table 1). This result was not expected, since SE counts were reduced when the high-threonine diet was supplemented with mannanoligossacharide (MOS) in a previous study carried out in our laboratory [9]. On the other hand, intestinal integrity was improved in the present study in those chicks fed high threonine levels and inoculated with SE, including higher villus height and villus:crypt ratio. Thus, we believe that higher dietary levels of threonine somehow compensate for the increased demands on mucus production due to the challenge. Chicks are susceptible to many and diverse pathogens during the first weeks of life, a period during which the immune system is not fully developed [30], and depend basically on two defense mechanisms, the innate immune system, including mucus and phagocytes, and the presence of maternal antibodies [31]. Although maternal antibodies are considered the main protection mechanism of hatchlings [30,32], the mucus layer in the intestines is crucial. It not only serves as substrate for the adherence of commensal bacteria, but also helps preventing infection by pathogenic bacteria and the resultant mucosa injury [33,34]. Mucus is constituted by 95% of water and 5% of mucins, glycoproteins particularly rich in threonine [35]. It has been previously suggested that threonine is closely related to intestinal protection [36]. During stress, particularly inflammation, threonine is highly retained by the intestines in order to maintain the integrity and function necessary in the intestine [7] and mucin synthesis in piglets and rats [10,37] is impaired in case threonine levels are limited. The protection barrier is thus compromised, not only by the fewer mucin present, but also by the changes in mucin characteristics, which might result in significant functional changes [10].

Mucus is secreted by goblet cells and changes in goblet cell numbers may be related to sanitary challenges that require greater mucus production [1]. Indeed, in the present study, there was an increase in goblet cell counts in the jejunum and ileum of inoculated chicks (Table 3 and S2 Table), either heat-treated or fed high levels of threonine, when compared with sham-inoculated birds. This may be related, firstly, to the fact that these segments need more threonine and, secondly, because these are preferred sites of microbial establishment [11]. This behavior was not observed in the duodenum; sham-inoculated and inoculated birds had similar counts. Amongst the intestinal segments, duodenum does not offer favorable conditions for the establishment of microbial populations due to the presence of numerous enzymes, high oxygen pressure, high concentrations of antimicrobial compounds such as bile salts, and the reflux movements from the jejunum to the gizzard that result in a rapid shift in the environment conditions [8]. Nevertheless, it should be noted that the smaller number of goblet cells in the duodenum of cold-treated birds might be related to the higher *Salmonella* counts and number of infected birds after the challenge in those animals.

The increased expression of the *muc2* gene observed in this study when threonine was fed at higher levels corroborates a recent report [11] attributing the direct interaction between threonine and goblet cells due to a linear increase in the expression of acid mucin gene (MUC2) in the ileum and jejunum of layers fed high threonine levels and submitted to heat stress.

Interestingly, the present data have shown that the challenge with SE was not able to cause injury to the epithelium when heat-treated birds or high-threonine fed birds were compared to sham-inoculated birds. The results corroborate Zaefarian et al. [38], who reported a positive effect of threonine supplementation on villus height, epithelial thickness, number of goblet cells and crypt depth in the three segments of the small intestine. Bacteria rely on mechanisms of mucolysis, among others, to penetrate the mucus layers and eventually invade host cells [39]. It has been shown that inflammation increases both extrusion at the villus apex and crypt hyperplasia [40], and the life span of enterocytes, which is usually 48 to 96 hours [41], may be decreased. Situations such as the pathogen challenge model used in the present study raise even further the already elevated costs of mucosa maintenance; not only an immune response must be mounted, but also epithelium renewal and crypt cell multiplication are accelerated, resulting in villus atrophy and hyperplasia of crypt [42]. Furthermore, digestion and nutrient absorption are affected and performance is compromised. Therefore, it also our belief that maintaining the integrity of the intestinal epithelium will help prevent *Salmonella* infection.

The maturation of the gut immune system has been shown to be dependent on the age of birds and the interaction of host cells and the microbiota that is being established [43] and the defense systems might act synergistically [44]. Since broiler chicks have not contact with adult birds and therefore their microbiota in order to help colonizing their gut [43], future studies should focus on how the manipulation of the diet or the incubation temperature might be used to improve gut health and to make birds more capable of coping with potential pathogens in the very first days post-hatch.

In conclusion, either high temperature during incubation or high threonine levels in the diet improve intestinal integrity and mucus production, and decrease stress response, reflecting on better body weight and weight gain at 8dpi (10 days of age), which were similar to sham-inoculated birds. Possibly, different mechanisms may be responsible for the response in each case, since high incubation temperature decreased both the number of SE-infected birds and SE counts, what was not seen in birds fed higher threonine level. On the other hand, low incubation temperature was not able to protect from infection or to reduce damage to the intestinal epithelium, and the birds showed impaired performance after inoculation with SE. These findings encourage further studies involving more holistic approaches to investigate the epigenetic effects of incubation conditions on chicks’ resistance against pathogenic agents.

## Supporting Information

S1 Table Composition and nutritional levels of experimental diets. (DOCX)

S2 Table Effects of embryonic thermal manipulation and threonine levels on duodenum and jejunum morphometry (villus height, crypt depth and villus: crypt ratio; n = 120) and goblet cell counts (n = 12) of chicks inoculated with *Salmonella* Enteritidis (8 dpi). (DOCX)

S3 Table Effect of embryonic thermal manipulation on incubation parameters and weight of chicks at hatch. (DOCX)
