# Supplementary material for: High Incubation Temperature and Threonine Dietary Level Improve Ileum Response Against Post-Hatch Salmonella Enteritidis Inoculation in Broiler Chicks
Source: PLoS One. 2015 Jul 1;10(7):e0131474. doi: 10.1371/journal.pone.0131474 (PMC4488937; doi:10.1371/journal.pone.0131474)
Supplement: S1 Table — (DOCX) [file pone.0131474.s001.docx]

**S1 Table. Composition of feed.**

|  | Threonine level | | | |
| --- | --- | --- | --- | --- |
|  | 1 to 7 d | | 7 to 10 d | |
| Ingredients | Basal | High | Basal | High |
| Corn | 57.56 | 57.56 | 58.00 | 58.00 |
| Soybean meal (45%) | 27.30 | 27.30 | 26.00 | 26.00 |
| Gluten meal (60%) | 6.50 | 6.50 | 6.35 | 6.20 |
| Dicalcium phosphate | 1.95 | 1.95 | 1.55 | 1.55 |
| Soybean oil | 3.50 | 3.50 | 4.10 | 4.10 |
| Limestone | 0.85 | 0.85 | 0.88 | 0.87 |
| Salt | 0.40 | 0.40 | 0.35 | 0.35 |
| DL-Methionine | 0.30 | 0.30 | 0.24 | 0.23 |
| L-Lysine-HCl | 0.57 | 0.57 | 0.48 | 0.47 |
| L-Threonine | 0.15 | 0.27 | 0.10 | 0.20 |
| Sand | 0.77 | 0.65 | 1.80 | 1.88 |
| Choline chloride | 0.07 | 0.07 | 0.07 | 0.07 |
| Mineral premix1 | 0.05 | 0.05 | 0.05 | 0.05 |
| Vitamin premix2 | 0.03 | 0.03 | 0.03 | 0.03 |
| Total | 100.00 | 100.00 | 100.00 | 100.00 |
| Nutrient levels |  |  |  |  |
| Metabolizable energy (kcal/kg) | 2,950 | 2,943 | 3,002 | 3,001 |
| Crude protein (%) | 22.17 | 22.16 | 20.80 | 20.79 |
| Digestible threonine (%) | 0.857 | 0.956 | 0.764 | 0.852 |
| Calcium (%) | 0.921 | 0.917 | 0.821 | 0.818 |
| Available phosphorus (%) | 0.470 | 0.469 | 0.390 | 0.390 |

^1^Mineral premix (concentration/kg product): Mn - 60 g, Fe – 80 g Zn - 50 g Cu - 10 g Co - 2 g, I - 1 g; Se - 250 mg. ^2^Vitamin premix (concentration/kg product): Vit. A – 15,000 IU, Vit. D3 - 1,500,000 IU. Vit. E – 15,000 IU; Vit.B1 - 2.0 g, Vit. B2 - 4.0 g Vit. B6 - 3.0 g, Vit. B12 – 0.015 g, nicotinic acid - 25 g, Pantothenic acid - 10 g; Vit.K3 - 3.0 g, folic acid - 1.0 g.
