# Supplementary material for: High Incubation Temperature and Threonine Dietary Level Improve Ileum Response Against Post-Hatch Salmonella Enteritidis Inoculation in Broiler Chicks
Source: PLoS One. 2015 Jul 1;10(7):e0131474. doi: 10.1371/journal.pone.0131474 (PMC4488937; doi:10.1371/journal.pone.0131474)
Supplement: S2 Table — (DOCX) [file pone.0131474.s002.docx]

**S2 Table. Effect of embryonic thermal manipulation and threonine levels on duodenum and jejunum morphometry (villus height, crypt depth and villus: crypt ratio, n=120) and goblet cell counts (n=12) of chicks inoculated with *Salmonella* Enteritidis (8 dpi).**

| Duodenum |  | Villus height (V, µm) | Crypt depth (C, µm) | V:C ratio | Goblet cell counts (n) |
| --- | --- | --- | --- | --- | --- |
| Incubation temperature | Low (36.7ºC) | 1362.3 ± 123.6 bB | 126.9 ± 19.4 aA | 11.1 ± 1.7 bB | 86.8 ± 21.2 bB |
|  | Standard (37.7ºC) | 1352.4 ± 130.6 bB | 121.8 ± 10.4 bA | 10.7 ± 1.5 bB | 108.3± 32.4 aA |
|  | High (38.7ºC) | 1546.4 ± 140.5 aA | 110.2 ± 15.3 cA | 15.8 ± 2.1 aB | 96.1 ± 25.1 abA |
|  | Sham-inoculated | 1401.8 ± 142.8 B | 73.1 ± 12.9 B | 19.7 ± 2.1 A | 116.3 ± 18.9 A |
| Threonine level | Basal (0.857%) | 1379.6 ± 141.1 aA | 125.5 ± 15.8 aA | 12.5 ± 3.0 aB | 81.0 ± 22.9 bB |
|  | High (0.956%) | 1390.9 ± 146.1 aA | 113.8 ± 14.9 bA | 12.9 ± 2.7 aB | 113.9 ± 19.8 aA |
|  | Sham-inoculated | 1401.8 ± 142.8 A | 73.1 ± 12.9 B | 19.7 ± 2.1 A | 116.3 ± 18.9 A |
| Jejunum |  |  |  |  |  |
| Incubation temperature | Low (36.7ºC) | 735.0 ± 73.0 bB | 97.4 ± 12.3 aA | 7.5 ± 0.6 bB | 134.4 ± 17.9 bA |
|  | Standard (37.7ºC) | 744.6 ± 41.9 bB | 98.2 ± 12.8 aA | 7.7 ± 0.7 bB | 149.0 ± 20.2 aA |
|  | High (38.7ºC) | 839.0 ± 65.9 aA | 90.6 ± 8.6 bA | 9.3 ± 0.4 aA | 151.3 ± 20.8 aA |
|  | Sham-inoculated | 825.0 ± 116.1 A | 61.8 ± 8.6 B | 13.4 ± 0.5 A | 121.3 ± 9.1 B |
| Threonine level | Basal (0.857%) | 774.7 ± 76.9 aB | 100.0 ± 12.5 aA | 7.9 ± 1.0 bB | 133.0 ± 20.2bB |
|  | High (0.956%) | 771.1 ± 63.6 aB | 90.7 ± 9.6 bA | 8.6 ± 0.6 aB | 156.8 ± 15.1aA |
|  | Sham-inoculated | 825.0 ± 116.1 A | 61.8 ± 8.6 B | 13.4 ± 0.5 A | 121.3 B |

Within each factor, means followed by the same small letter in the column are similar by Tukey’s test (5%).

Within each factor, means followed by the same capital letter in the column are similar to the sham-inoculated treatment by Dunnet’s test (5%).
