# Supplementary material for: High Incubation Temperature and Threonine Dietary Level Improve Ileum Response Against Post-Hatch Salmonella Enteritidis Inoculation in Broiler Chicks
Source: PLoS One. 2015 Jul 1;10(7):e0131474. doi: 10.1371/journal.pone.0131474 (PMC4488937; doi:10.1371/journal.pone.0131474)
Supplement: S3 Table — (DOCX) [file pone.0131474.s003.docx]

**S3 Table. Effect of embryonic thermal manipulation on incubation parameters and weight of chicks at hatch.**

|  |  | Incubation Temperature | | |
| --- | --- | --- | --- | --- |
|  |  | Low (36.7ºC) | Standard (37.7ºC) | High (38.7ºC) |
|  | Number of eggs (n) | 80 | 80 | 80 |
|  | Fertile eggs, % (fertile/total eggs) | 86.3 (69/80) | 85.0 (68/80) | 95.0 (76/80) |
| Incubation parameters | Total hatchability, % (hatchlings/total eggs) | 75.0 (60/80) | 73.75 (59/80) | 87.5 (70/80) |
|  | Fertile hatchability, % (hatchlings/fertile eggs) | 86.9 (60/69) | 86.7 (59/68) | 92.1 (70/76) |
| Mortality during incubation | Total mortality, % (dead/total fertile eggs) | 13.04 (9/69) | 13.23 (9/68) | 7.89 (6/76) |
|  | 1st week, % (n, total dead) | 0 | 0 | 0 |
|  | 2nd week, % (n, total dead) | 22.2 (2/9) | 0 | 33.3 (2/6) |
|  | 3rd week, % (n, total dead) | 77.8 (7/9) | 100 (9/9) | 66.7 (4/6) |
| At hatch | Body weight (g) | 50.6 | 50.3 | 50.9 |
